# From Chloroplast Biogenesis to Chlorophyll Accumulation: The Interplay of Light and Hormones on Gene Expression in *Camellia sinensis* cv. Shuchazao Leaves

**DOI:** 10.3389/fpls.2020.00256

**Published:** 2020-03-11

**Authors:** Linlin Liu, Ning Lin, Xuyang Liu, Shu Yang, Wei Wang, Xiaochun Wan

**Affiliations:** State Key Laboratory of Tea Plant Biology and Utilization, International Joint Laboratory on Tea Chemistry and Health Effects of Ministry of Education, Anhui Agricultural University, Hefei, China

**Keywords:** *Camellia sinensis*, chlorophyll, chloroplast, hormones, light regulation, response to shade

## Abstract

Chloroplast development and chlorophyll metabolism have been well described in model plants but not in perennial woody crops. Of particular interest is the interplay between light and hormones under shade conditions. We report that the shade induced accumulation of chlorophylls in *Camellia sinensis* cv. Shuchazao leaves is at least as a result of (a) positive changes in chloroplast development and (b) light/hormonal regulation of genes and transcription factors involved in the chlorophyll biosynthesis pathway. Under shade conditions, leaves developed an abundance of enlarged chloroplasts encapsulating more prominent thylakoid membranes. Four major metabolites in the chlorophyll biosynthesis pathway namely Chl *a*, Chl *b*, DPP, and Mg-Proto IX increased under shade conditions while PBG decreased significantly. Significant changes were found at the transcription level of regulators of chloroplast biogenesis (*GLK1* and *LHCB*), the structural genes in the chlorophyll biosynthesis pathway (*HEMA1*, *CLH1*, *PORA*, and *CAO*) and potential components involved in light signaling (*PHYA*, *CRY1*, *HY5*, and *DELLAs*). Two central signal integrators (*GLK1* and *LHCB)* between the nucleus and chloroplast showed clear responses to shade, suggesting a crucial role of light in regulating chloroplast development in tea leaves. Concurrent with the changes in gene expression, the concentrations of endogenous phytohormones (auxin, cytokinin, and gibberellins) increased significantly in the later stages of shade conditions. Two key integrators involved in the hormone signal pathways, EIN3 and EBF1/2, increased under shade conditions suggesting that shade induced changes to hormone levels may play some role in modulating chlorophyll biosynthesis in the tea leaves. Overall, this data suggests that the light and hormone influence over chloroplast development and chlorophyll biosynthesis in *Camellia* is similar to that of *Arabidopsis*. This study provides new insights into the molecular mechanisms that regulate chlorophyll biosynthesis in response to light and hormones in a commercially important woody plant such as *Camellia*, which may facilitate the breeding of high-chlorophyll tea cultivars for the improvement of sensory features of the green tea product.

## Introduction

Tea, one of the products made from the processed leaves of tea plants [*Camellia sinensis* (L.) O. Kuntze], has been recognized as the most popular non-alcoholic beverage worldwide (Liu S. et al., 2018) because of the pleasant tastes, fragrances and health benefits (Chacko et al., 2010; Song et al., 2018). The color of the infusion is one of the most important characteristics determining the quality of the product (Kaneko et al., 2006) and been the focus of the tea industry. Green tea makes use of unwilted leaves and buds, and many strategies have been implemented to enhance and improve the color of tea products from the tea plantation to the processing factory. One such strategy is growing plants under shade. This improves the quality of fresh leaves through the enhancement of chlorophyll accumulation and the balancing of secondary metabolites (Song et al., 2012; Wang et al., 2012; Liu L. et al., 2018). The leaves of tea plants grown under shade conditions contain a higher level of chlorophylls (Liu G. F. et al., 2017; Liu L. et al., 2018), which contributes to a bright green color of the product (Wang et al., 2012). In addition to color improvement of the tea infusion, the shaded leaves contain high levels of amino acids but lower catechin content, which also improves the sensory qualities (Wang et al., 2012; Lu et al., 2014). Studies on the effect of shade on the tea plants were initiated decades ago, with major systematic analyses focused on the influence of secondary metabolites, such as catechin and flavonol biosynthesis (Koretskaya and Zaprometov, 1975; Saijo, 1980; Song et al., 2012). However, little attention has been paid to the mechanisms of regulation of chlorophyll biosynthesis by light when referring to the interplay between light and hormones. Under shade conditions when the roles of light and hormones are considered, the molecular mechanisms involved in chlorophyll biosynthesis underlying shading effects become more complex and intricate in this commercially important beverage crop.

In higher plants, chlorophylls are the most abundant tetrapyrroles. They function as photosynthetic pigments to harvest light energy and transfer the absorbed energy to the reaction center in which charge separation occurs (Tanaka and Tanaka, 2007; Tanaka et al., 2011). Chlorophyll is initially biosynthesized from glutamate, which is then converted to 5-aminolevulinic acid (ALA) and further converted to protochlorophyllide (DPP), leading to the “chlorophyll cycle” which refers to the interconversion between chlorophyll *a* (Chl *a*) and chlorophyll *b* (Chl *b*) (Tanaka and Tanaka, 2007; Tanaka et al., 2011). Chlorophyll metabolism in the model plant *Arabidopsis* has been well described, including all enzymatic pathways, encoding genes and intracellular location of enzymes, as well as the potential transcription factors (TFs) (Tanaka and Tanaka, 2007; Hörtensteiner and Kräutler, 2011; Tanaka et al., 2011; Liu X. et al., 2017). In contrast, reports on the activities of genes and enzymes of the chlorophyll biosynthesis pathway in perennial woody plants are still limited, particularly in relation to the chlorophyll metabolism modulated by light and hormones in *Camellia*.

Light is probably one of the most important environmental factors that regulate the chlorophyll biosynthesis pathway in higher plants (Tanaka and Tanaka, 2007). Red and far-red light sensors (phytochromes, PHYs), blue light receptors (cryptochromes, CRYs) and the circadian clock machinery mediate the light signaling, which modulates the expression of genes involving in chlorophyll metabolism (Tanaka et al., 2011). Two classes of TFs, namely Phytochrome Interacting Factors (PIFs) and Elongated Hypocotyl 5 (HY5), regulate chlorophyll biosynthesis in opposing manners (Chen et al., 2004; Leivar and Quail, 2011). PIFs, which accumulate in dark-grown seedlings and are directly targeted by photo-activated PHYs for degradation, negatively regulate chlorophyll biosynthesis and photosynthetic genes to optimize the greening process of the seedling (Stephenson et al., 2009; Song et al., 2014). Conversely, HY5 regulates nuclear gene transcription and functions as a central downstream factor in light signaling to promote seedling photomorphogenesis (Chen et al., 2004; Xu et al., 2016). While HY5 and PIF proteins act downstream of phytochrome signaling, the transcription factors Golden2-Like (GLK) 1 and GLK2 influence chlorophyll biosynthesis independently of the PHYB signaling pathway (Waters et al., 2009). GLKs strongly up-regulate the expression of genes that are involved in chlorophyll biosynthesis, including *Glu-tRNA reductase* (*HEMA1*), *magnesium chelatase* (*CHLH*), *pchlide oxidoreductase* (*PORB*), and *chlide a oxygenase* (*CAO*).

In addition to the influence of light, endogenous phytohormones are also recruited to mediate the metabolism of the chlorophyll biosynthesis pathway (Liu X. et al., 2017). Key components in the light signaling pathway such as HY5 and PIFs, connect light signals to the signaling pathways of multiple phytohormones, including cytokinin (CTK), gibberellin (GA) and auxin (Lau and Deng, 2010). It has been suggested that the two hormones (CTK and GA) serve to regulate HY5 at the protein level and that HY5 promotes photomorphogenesis partly by modulating auxin, GA and abscisic acid signaling (Lau and Deng, 2010). Ethylene (ETH) and its precursor (1-aminocyclopropane-1-carboxylate acid, ACC) play critical roles in leaf senescence, root elongation and protecting cotyledons from photooxidative damage when the seedlings are exposed to light (Liu X. et al., 2017; Han et al., 2019; Lv et al., 2019). Ethylene Insensitive 3/EIN3-Like 1 (EIN3/EIL1) is the master transcription factor in the ethylene signaling pathway (Guo and Ecker, 2004), which markedly inhibits the accumulation of pchlide and directly binds to the promoters of *PORA* and *PORB* to activate their gene expression (Zhong et al., 2014).

The biogenesis of chloroplasts is also subjected to light and hormone regulation (Waters and Langdale, 2009; Pogson et al., 2015). In dicotyledonous seedlings, chloroplast biogenesis can be described as the differentiation from the plastid progenitor to an undeveloped proplastid, a precursor structure that has no photosynthetic capacity, to a mature chloroplast (Kessler and Schnell, 2009; Waters and Langdale, 2009; Pogson et al., 2015). This process is highly light-dependent, differs between species and to date the molecular intricacies have not been fully characterized. Golden2-like proteins (GLK1 and GLK2) are an important class of nuclear transcription factors required for transcription of genes encoding chloroplast proteins, providing the anterograde signal from the nucleus to the chloroplast to trigger the induction of photomorphogenesis (Oh and Montgomery, 2014). Conversely, this forward communication is balanced by a retrograde signal from the chloroplast to the nucleus (Chi et al., 2015). A number of experimental approaches over the past 30 years have confirmed the existence of this retrograde signal and arrangement of integrators, which participate in this signal have been suggested to be light-regulated, such as HY5 (Chi et al., 2015) and the light-harvesting complex II chlorophyll binding protein (LHCB) (Waters and Langdale, 2009).

Evidence from previous studies indicates that the reduction of light exposure enhances chlorophyll accumulation in tea leaves (Song et al., 2012; Wang et al., 2012; Liu L. et al., 2018). However, the molecular mechanisms for chlorophyll biosynthesis are still mysterious, especially when referring to the interplay between light and hormones under shade conditions. It is unclear if the regulatory mechanisms of model plants and perennial woody plants such as the *Camellia* are similar. In recognition of the importance of chlorophyll content in determining green tea characteristics, we investigated the effect of shade conditions on chloroplast development, accumulation of major metabolites and the activities of genes/TFs involved in the chlorophyll biosynthesis pathway, and the potential roles of hormones in shade induced chlorophyll changes in tea leaves. To examine the effect of shade on chloroplast development and chloroplast ultrastructure, we measured the expression of genes/TFs that are involved in chloroplast biogenesis and differentiation. The levels of major metabolites in the chlorophyll biosynthesis pathway, including Chl *a*, Chl *b*, ALA, porphobilinogen (PBG), protoporphyrin IX (Proto IX), uroporphyrinogen III (Urogen III), DPP and Mg-Protoporphyrin IX (Mg-Proto IX) were also determined. Furthermore, we analyzed endogenous phytohormones likely to be involved in chloroplast development, including auxin, CTK, GA, brassinosteroid (BR), strigolactones (SL), and ACC. In addition, genes and TFs involved in chloroplast development and chlorophyll biosynthesis were analyzed at the transcriptional level, with emphasis on genes/TFs which potentially function in the regulation by light and or hormone on chlorophyll biosynthesis, as suggested by previous studies. Overall, this research aimed to investigate the interplay of light and hormones on the regulation of chlorophyll biosynthesis in response to shade conditions in a commercially important tea crop, *C. sinensis* cv. Shuchazao.

## Materials and Methods

### Shade Treatments and Sample Collection

Shade treatments and sample collections were described previously with minor modifications (Liu L. et al., 2018). This study was carried out at Anhui Agricultural University research tea plantation (31°.55′ North, 117°.12′ East; Tea Plant Cultivar and Germplasm Resource Garden in Guohe Town), using 24 years old *C. sinensis* cv. Shuchazao plants propagated from cuttings (Zhang X. et al., 2018). The plants were 1.4 m wide, 1.5 m tall from the soil surface and 0.5 m between plants within the row. 12 rows of plants (50 m long and 1.4 m wide of each row, 2 m between and 0.6 m within row spacing) were selected for the treatments. Treatments consisted of plants exposed to natural unobstructed light (control, C) and plants exposed to 80–90% shade (shade, S; 10–20% light transmitted). The black nylon shade nets (Non-gfeng Company, Hefei, China) were placed about 1.5 m above the tea plants on the12th of April in 2017 at the onset of a new round of budburst. Each treatment was replicated three times and the positions of treatments randomized statistically within 6 rows of plants.

Photosynthetically Active Radiation (PAR) was measured using a Light Scout^®^ Quantum Light Meter (Item#3415F, Spectrum Technology^®^ Inc., United States). For sample collections, tea buds with the first developing leaf were collected for RNA-Seq and RT-PCR analyses at different stages during shade treatments (4h, 1d, 3d, 7d, and 14d). For metabolite and hormone analysis, the first developing leaf was collected at different stages during shade treatments (1d, 3d, 7d, and 14d). For chloroplast ultrastructure observation, the first developing leaf was collected at 21d of shade treatment. All the materials were frozen immediately in the field using liquid nitrogen and stored at −80°C for future analysis.

### Chloroplast Ultrastructure

Fresh leaves were excised and immediately dipped in pre-cooled glutaraldehyde PBS solution (5% glutaraldehyde in 0.1 M PBS, pH 7.2, 4°C), assisted by a syringe for better infiltration according to a previous publication (Liu G. F. et al., 2017). Glutaraldehyde-infiltrated leaves were cut into small pieces (about 2 mm × 3 mm) and sectioned on a TCS CM1900 freezing microtome (Leica, Germany). The ultrathin sections were double lead stained and photomicrographed using an HT-7700 transmission electron microscope (TEM; Hitachi, Japan). The preparation of the ultrathin section, staining, and TEM observation was carried out at the Biotechnology Center in Anhui Agricultural University (Hefei, China).

### Metabolite and Phytohormone Analysis

Chlorophyll analysis was carried out as described previously (Lu et al., 2019). Fresh leaves (0.1 g) were cut into small pieces and extracted overnight in 10 ml solvent (5% acetone in 95% ethanol, v/v) until the pieces became completely decolored. Absorbance of the extract was measured in an ultraviolet spectrophotometer (U-5100, Hitachi, Japan) at A_645_ and A_663_ and the concentrations chlorophyll *a* and *b* were calculated using the formula: Chl *a* = 12.7A_663_ – 2.69A_645_; Chl *b* = 22.9A_645_ – 4.68A_663_ (Arnon, 1949).

The concentrations of major metabolites in the chlorophyll biosynthesis pathway and phytohormones, including ALA, PBG, Urogen III, Proto IX, Mg-Proto IX, DPP, CTK, GA, ACC, BR, and SL were determined using enzyme-linked immunosorbent assay (ELISA) kits (mIbio Co. Ltd., Shanghai, China) according to the manufacturer as reported previously (Liu et al., 2019). The principle of the kit is based on the original ELISA method with modifications. Purified plant antibody was used to coat microtiter plate wells to create a solid-phase antibody. Once the selected compound was added into the wells, the compound was combined with the second antibody which was labeled conjugated to a specific enzyme, and then the antibody-antigen-enzyme complex was incubated. The concentration of the ligand was further determined according to the following enzymatic chromogenic reaction. Tea leaves were collected and grounded in liquid nitrogen. Accurately 100 mg of the powder was weighed and extracted in a fixed volume of 0.01 M PBS buffer (pH 7.4; PBS: tissue ratio = 10: 1, v/w) overnight at 4°C. Then the suspension was centrifuged at 10,000 rpm for 10 min and the supernatant was collected for further determination. Forty microliter of the Sample Dilution was added into the well of the Microelisa Stripplate (blank well did not add sample and the HRP-Conjugate reagent, wells for the standard curve followed the same operation), and 10 μl of the supernatant was added into the sample well (final dilution is fivefold), then mixed gently by shaking the plate. Subsequently, 100 μl of the HRP-Conjugate Reagent was added into each well (except the blank well), then the plate was sealed with the Closure Membrane and incubated at 37°C for 60 min. Then the Closure Membrane was removed, the reagent was discarded and the well was washed 5 times by the Wash Solution. After drying in a fume hood for 5 min, 50 μl of the Chromogen Solution A and 50 μl of the Chromogen Solution B were added into each well, then the plate was placed in the dark for 15 min at 37°C and the reaction was stopped by 50 μl of the Stop Solution. The absorbance at 450 nm was detected within 15 min of adding the stop solution and the concentration of the compound was calculated by extrapolation from the standard curve and the concentrations expressed per mass of leaf tissue extracted.

### RNA-Seq Analysis

RNA-Seq analysis was conducted as described previously (Liu L. et al., 2018). RNA extraction, library construction and RNA-Seq performed by the Illumina HiSeq2000 were carried out by Wuhan Bosaixi Biotechnology Company (Wuhan, China). Clean reads were combined and separately assembled using the transcriptome assembler Trinity (version r20140717). After removing adaptor sequences, duplication sequences, ambiguous reads and low-quality reads, an average of 6 GB clean data per sample was generated. The final assembly of tea samples had 82322 unigenes (≥500 bp) with an N50 length of 1,206 bp. Functional annotation revealed 57823, 40003, 34066, 11963, and 34972 unigenes with alignments to NR (non-redundant protein database), Swiss-Prot (Annotated protein sequence database), KOG (Clusters of orthologous groups for eukaryotic complete genomes), KEGG (Kyoto encyclopedia of genes and genomes) and GO (Gene ontology) databases, respectively. Selected unigenes were validated according to the tea plant genome in the Tea Plant Information Archive (TPIA) (Wei et al., 2018; Xia et al., 2019)^1^.

### RT-PCR Analysis

RT-PCR analysis was conducted as previously described with minor modifications (Tai et al., 2015). Total RNA was extracted using the Spectrum^TM^ Plant Total RNA Kit (Sigma-Aldrich, Shanghai, China). TURBO DNA-free^TM^ Kit (Sigma-Aldrich, Shanghai, China) was used to remove traces of genomic DNA. Single-stranded cDNA used for RT-PCR were synthesized using a Prime-Script^TM^ Strand cDNA Synthesis Kit (Takara, Dalian, China). The SYBR green method (Takara, Dalian, China) was used for RT-PCR analysis performed by a CFX96 Touch real-time PCR detection system (Bio-Rad, United States). Tea gene *CsACTIN* (HQ420251.1) was used as an internal housekeeping gene. The relative expression levels of the amplified products were calculated using the 2^–ΔΔCt^ method (Yang and Huang, 2018). The primers for selected unigenes were designed online by NCBI Primer-BLAST (Supplementary Table S1)^2^.

### Statistic Analysis

All data presented in this study were calculated from three biological replicates, including the concentrations of chlorophylls and metabolites, hormones, RNA-Seq and RT-PCR analyses. Statistical analyses were conducted using the Minitab 17.0 statistical software (Minitab Inc., Coventry, United Kingdom). Data were analyzed by one-way analysis of variance (ANOVA) and a Fisher’s least significant difference (LSD) test at the 5% level.

## Results

### Shade Induced Stimulation of Chloroplast Development

**FIGURE 1:**
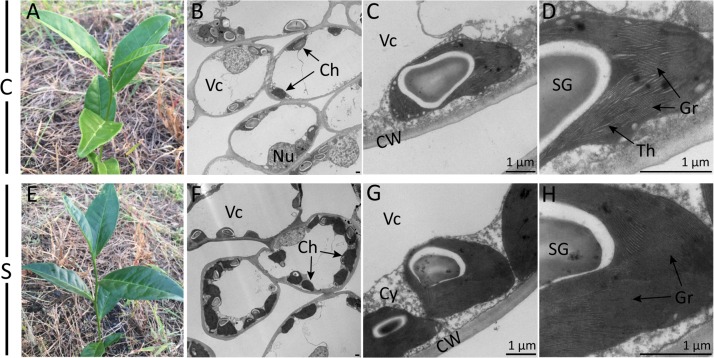
Effect of shade on *camellia* leaves at the gross- and ultrastructural levels. Gross images show the coloration of typical control **(A)** and shaded **(E)** tea shoots. Photomicrographs show the ultrastructure of chloroplasts in both the control treatment **(B–D)** and the shade treatment **(F–H)** collected at 21 days of treatment. Magnifications of the TEM images were captured at ×0.7K **(B,F)**, ×5K **(C,G)** and ×12K **(D,H)** on a HT-7700 transmission electron microscope (Hitachi, Japan). Lead stain. C, control treatment; S, shade treatment; Vc, vacuole; Ch, chloroplast; Nu, nucleus; CW, cell wall; Cy, cytosol; SG, starch granule; Th, thylakoid; Gr, grana. Bars = 1 μm.

Visual changes in leaf color traits were observed after shade treatment (Figure 1). An enhancement of dark green color could be observed visually from the second day to 2 weeks of shade. TEM observation further revealed dramatic responses to shade in chloroplast development. When compared to the non-shaded mature leaves of the control treatment, in shaded leaves, chloroplasts were (a) more numerous (8.67 per cell transection compared to 3.25 per cell transection) and (b) more rounded (Figures 1B,F). The number of starch granules per cell transaction increased due to the higher number of the chloroplast. Furthermore, the shaded leaves contained more tightly and intensively stained grana stacks, with a much higher number of thylakoids per granum and a higher stacking degree of thylakoids (Figures 1C,D,G,H).

### Shade Induced Changes to Chlorophyll and Metabolite Concentrations

**FIGURE 2:**
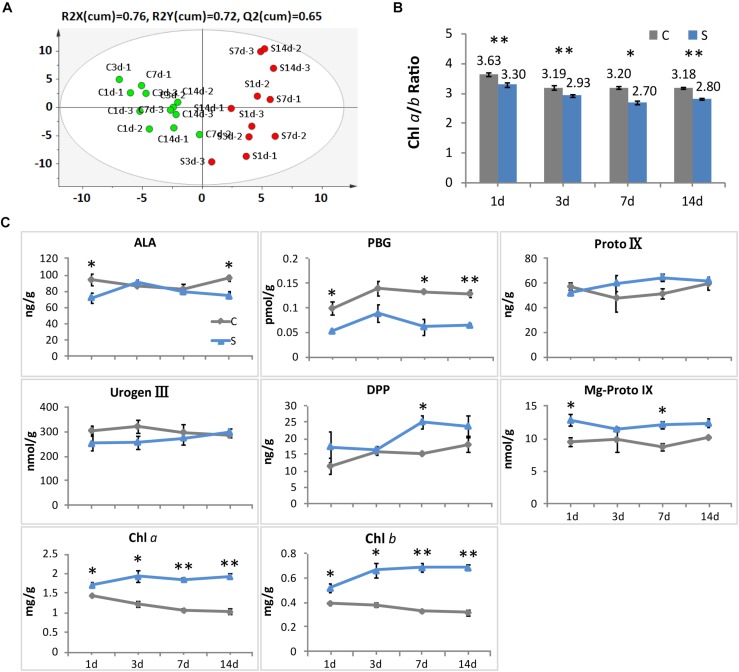
Effect of shade on the concentrations of chlorophylls and the main precursors in tea leaves. **(A)** The OPLS-DA analysis of the main precursors and chlorophylls in tea leaves from the control and shade treatments. **(B)** The ratio of chlorophyll *a* and chlorophyll *b* in tea leaves from the control and shade treatments. **(C)** The accumulation of chlorophylls and main precursors in tea leaves from the control and shade treatments. 4h, 1d, 3d, 7d, and 14d indicate time points during the shade period. ALA, 5-aminolevulinic acid; PBG, porphobilinogen; Proto IX, protoporphyrin IX; Urogen III, uroporphyrinogen III; DPP, divinyl protochlorophyllide; Mg-Proto IX, Mg-Protoporphyrin IX; Chl *a*, chlorophyll *a*; Chl *b*, chlorophyll *b*. Data shown are from three independent biological replicates (*n* = 3). ^∗^Significant differences comparing the control treatment at each time point according to one-way ANOVA test and a Fisher’s LSD at the 5% significance level (^∗^*p* ≤ 0.05, ^∗∗^*p* ≤ 0.01). OPLS-DA analysis was conducted by SIMCA 13.0 (UMETRICS, https://umetrics.com/).

Consistent with the alteration in leaf color, significant responses to shade conditions were detected in the concentrations of major metabolites in the chlorophyll biosynthesis pathway in tea leaves (Figure 2). The concentrations of Chl *a* and Chl *b* were significantly increased in shaded leaves, which were about twofold higher than that in the non-shaded leaves (Figure 2C). Simultaneously, a decrease was observed in the ratio of Chl *a*/*b* at different time points during shade treatment (Figure 2B). Among the major metabolites involved in the chlorophyll biosynthesis, DPP and Mg-Proto IX showed some increases in response to shade, while a significant decrease was found in the PBG accumulation. Proto IX and Urogen III were also analyzed, but no significant changes were found between the control and shade treatments.

### Shade Regulation of Genes Involved in Chloroplast Biogenesis

To investigate the effect of shade on chloroplast development at the transcriptional level, RNA-Seq technology was utilized to analyze gene activities in tea buds from both the control and shade treatments. The representative unigenes were selected from its multiple transcripts according to a comprehensive evaluation of parameters according to the following order: unigenes with best alignments to the reported sequence, the annotated unigenes with the highest fragment per kilobase of exon model per million mapped reads (FPKM), and unigenes differentially expressed.

**FIGURE 3:**
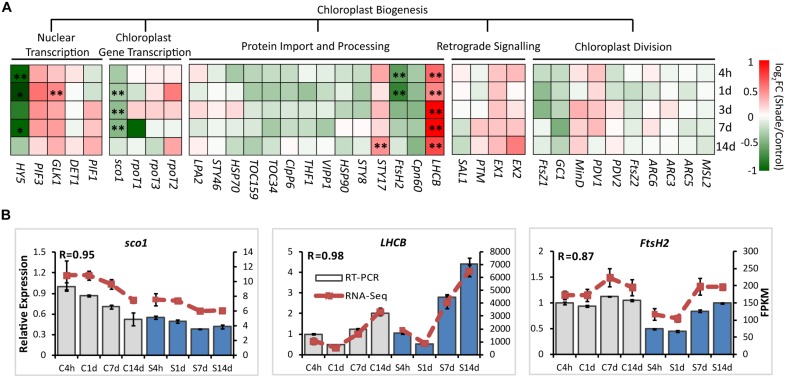
Effect of shade on genes encoding key enzymes of the chlorophyll biosynthesis pathway. **(A)** Transcript abundance of 38 representative unigenes involved in the chlorophyll biosynthesis pathway generated from RNA-Seq analysis. Genes and TFs are classed into different subgroups according to their functions involved in the chloroplast biogenesis, including nuclear transcription, chloroplast gene transcription, protein import and processing, retrograde signaling and chloroplast division. **(B)** Transcript abundance of selected unigenes validated by the RT-PCR analysis. The column presents data generated from the RT-PCR analysis (gray column, data from the control treatment at four time points; blue column, data from the shade treatment at four time points). The line presents data generated from the RNA-Seq analysis. The transcript abundance in the control treatment at 4 h (C4h) was set at 1. R, the correlation factor between RT-PCR and RNA-Seq analysis for each gene/TF according to SPSS 13.0 software. 4h, 1d, 3d, 7d, and 14d indicate time points during the shade period. C, control treatment; S, shade treatment; FPKM, Fragment per kilobase of exon model per million mapped reads. Data shown are conducted of three independent biological replicates (*n* = 3). ^∗^Significant differences comparing the control treatment at each time point according to one-way ANOVA and a Fisher’s LSD test at the 5% level (^∗^*p* ≤ 0.05, ^∗∗^*p* ≤ 0.01; fold change ≥ 1.5). For abbreviations, see text.

For chloroplast development, the transcript abundance of 37 annotated unigenes involved in the chloroplast biogenesis was analyzed. These candidate genes included genes involved in nuclear transcription, chloroplast gene transcription, protein import and processing, retrograde signaling and chloroplast division (Figure 3 and Supplementary Table S2). From 4h to 14d of shade, a group of unigenes showed significant increases. GLK1, the known nuclear transcript factor directly required for the transcription of genes encoding chloroplast proteins for chloroplast development (Waters et al., 2009), showed a consistent increase under shade conditions. LHCB was shown to directly interact with snowy cotyledon 2 (SCO2) which suggests a role in loading the vesicles with the photosynthesis-related proteins for transport to the thylakoids (Tanz et al., 2012). A dramatic increase of *LHCB* expression was found throughout the whole period of shade treatment. Conversely, *SCO2* presented a significant decrease in response to shade, especially during the early period of treatment. HY5, a transcription factor that has been reported to mediate light signaling to regulate nuclear gene transcription for chloroplast biogenesis in *Arabidopsis* (Xu et al., 2016), showed a significant decrease in gene expression throughout shade conditions. Similarly, the metalloprotease encoding gene *Filamentation temperature-sensitive H2* (*FtsH2*) showed decreases immediately at 4h and 1d, but no significant response was detected in the later period of shade treatment.

### Shade Regulation of Genes Involved in Chlorophyll Metabolism

**FIGURE 4:**
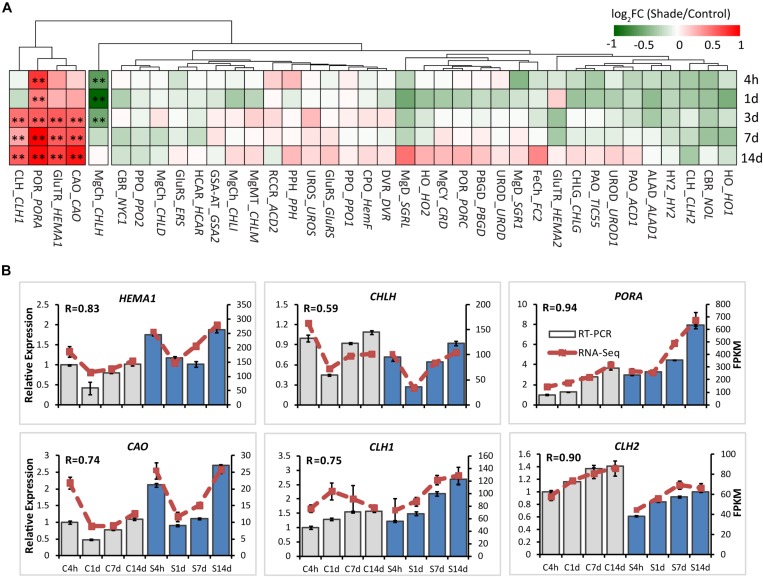
Effect of shade on key genes and TFs involved in chloroplast biogenesis. **(A)** Transcript abundance of representative unigenes involved in chloroplast biogenesis generated from RNA-Seq analysis. **(B)** Transcript abundance of selected unigenes validated by the RT-PCR analysis. The column presents data generated from the RT-PCR analysis (gray column, data from the control treatment at four time points; blue column, data from the shade treatment at four time points). The line presents data generated from the RNA-Seq analysis. The transcript abundance in the control treatment at 4 h (C4h) was set at 1. R, the correlation factor between RT-PCR and RNA-Seq analysis for each gene/TF according to SPSS 13.0 software. Data shown are conducted of three independent biological replicates (*n* = 3). The annotations of 4h, 1d, 3d, 7d, 14d, C, S, FPKM and statistical label (*) are shown as in Figure 3. For abbreviations, see text.

To explore the effects of shade on the activities of genes involved in chlorophyll metabolism, 38 annotated unigenes encoding 26 major enzymes were annotated from tea transcriptome datasets (Supplementary Table S3 and Figure 4). These candidate genes could be conceptually divided into several groups: 13 genes involved in the “common steps”; 8 genes involved in the “chlorophyll branch”; 7 genes involved in the “chlorophyll cycle”; 6 genes involved in the “chlorophyll breakdown”; and 4 genes involved in the “Heme/bilin branch.” Among these, genes involved in the “chlorophyll cycle” significantly increased in transcript level throughout the whole shade period. As shown in Figure 4, *chlorophyllase 1* (*CLH1*) and *CAO*, which participates in the conversion between Chl *a* and Chl *b*, showed a significant increase from 3d to 14d of shade. The transcript of *HEMA1* presented a similar response, with a significant increase at the later stage of shade treatment. *PORA* showed a significant early decrease after 4h and the transcript level increased at the later stage of shade treatment. In contrast, *CHLH*, which inserts Mg^2+^ into Proto IX, showed a significant decrease during the early stages (4h to 3d) of shade treatment but the expression level returned to normal after 14d.

### Shade Induced Changes to the Concentrations of Phytohormones

To determine the responses to shade on chloroplast development and chlorophyll accumulation in more detail, the concentrations of various phytohormones were analyzed in both the shaded and non-shaded leaves (Table 1). Six hormones were analyzed at different stages of shade treatment (1d, 3d, 7d, and 14d). CTK, which was reported to regulate the expression of many chloroplast-related genes (Chiang et al., 2012) and mediate the etioplast-to-chloroplast transition (Cortleven and Schmülling, 2015). CTK showed a significant increase in leaves under shade conditions. At day 1 the concentration had increased to 142% of the control and by days 3–123% of the control. Both GA and auxin were seen to increase in concentration later in the shade treatment period. The concentration of GA increased from 0.83 to 1.05 pmol/g after 14 days and increased by 42 and 45% of the control values at 7d and 14d of shade, respectively. It has been reported that ETH, BR and SL were involved in the hormone regulation of chloroplast development and photo-pigmentation accumulation in *Arabidopsis* (Zhong et al., 2009; Luo et al., 2010; Tsuchiya et al., 2010). No significant changes in ETH, BR and SL concentrations were detected and ACC showed only a slight but significant decrease at 3d of shade treatment.

**TABLE 1:** Leaf concentrations of endogenous phytohormones under control (C) and shade (S) conditions.

	CTK (ng/g)	GA (pmol/g)	Auxin (μmol/g)	ACC (μmol/g)	BR (ng/g)	SL (ng/g)
C1d	329 ± 3.4**	0.8 ± 0.1	0.10 ± 0.02	2.09 ± 0.31	42 ± 9.7	1090 ± 22.4
C3d	341 ± 53.5	0.8 ± 0.02*	0.11 ± 0.01	2.48 ± 0.02**	46 ± 2.2	878 ± 58.6
C7d	353 ± 24.4*	0.8 ± 0.01	0.12 ± 0.02*	1.79 ± 0.06	41 ± 9.6	1009 ± 51.7
C14d	377 ± 58.9	0.8 ± 0.1*	0.11 ± 0.02*	1.88 ± 0.04	39 ± 8.9	820 ± 34.5
S1d	469 ± 3.4**	0.8 ± 0.03	0.12 ± 0.01	1.85 ± 0.08	46 ± 11.9	909 ± 144.8
S3d	421 ± 13.8	1.0 ± 0.1*	0.13 ± 0.01	1.63 ± 0.15**	44 ± 4.7	880 ± 80.9
S7d	432 ± 6.6*	1.0 ± 0.1	0.17 ± 0.01*	1.96 ± 0.04	51 ± 5.4	1010 ± 29.3
S14d	447 ± 34.1	1.1 ± 0.1*	0.16 ± 0.02*	1.73 ± 0.14	45 ± 4.2	919 ± 72.4

1d, 3d, 7d, and 14d indicate time points during the shade period. CTK, cytokinin; GA, gibberellins; ACC, 1-aminocyclopropane carboxylic acid; BR, brassinosteroid; SL, strigolactones. Data shown are from the value of three independent biological replicates (n = 3). *Significant differences comparing the control treatment at each time point according to one-way ANOVA test and a Fisher’s LSD at the 5% significance level (*p ≤ 0.05, **p ≤ 0.01).

### Effect of Shade on the Activities of TFs and Integrators of the Light-Hormone Signal Networks

**FIGURE 5:**
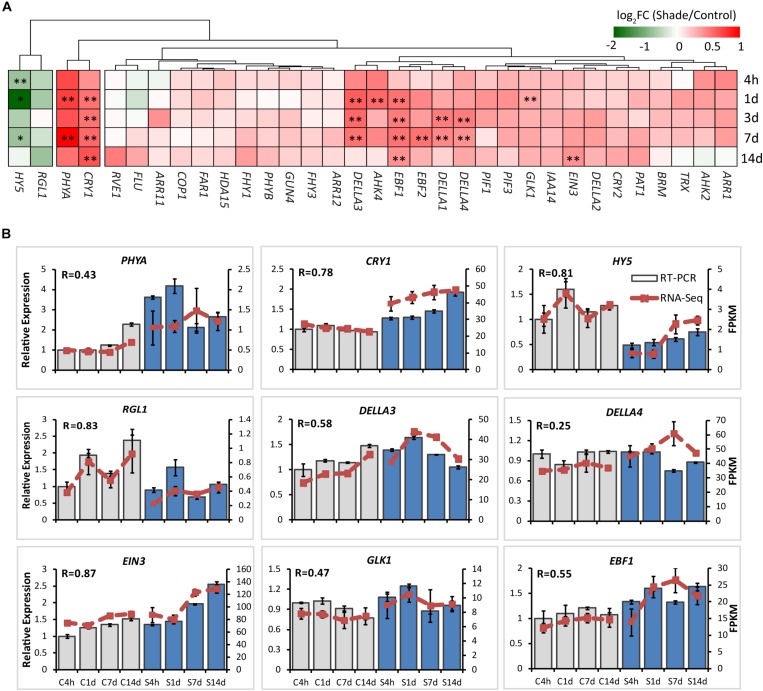
Effect of shade on key genes and TFs involved in the light- and hormone regulation of chlorophyll biosynthesis. **(A)** Transcript abundance of representative unigenes involved in the light and hormone regulation of the chlorophyll biosynthesis generated from RNA-Seq analysis. **(B)** Transcript abundance of selected unigenes validated by the RT-PCR analysis. The column presents data generated from the RT-PCR analysis (gray column, data from the control treatment at four time points; blue column, data from the shade treatment at four time points). The line presents data generated from the RNA-Seq analysis. The transcript abundance in the control treatment at 4 h (C4h) was set at 1. R, the correlation factor between RT-PCR and RNA-Seq analysis for each gene/TF according to SPSS 13.0 software. Data shown are conducted of three independent biological replicates (*n* = 3). The annotations of 4h, 1d, 3d, 7d, 14d, C, S, FPKM and statistical label (*) are shown as in Figure 3. For abbreviations, see text.

It has been well known that light and hormones play important roles in regulating chlorophyll accumulation in *Arabidopsis* (Lau and Deng, 2010; Liu X. et al., 2017). The integrators, such as PHYA, CRY1, PIFs, HY5, EIN3, and DELLA proteins, are key transcription regulators in light and/or hormone signaling pathways (Tanaka et al., 2011; Liu X. et al., 2017). Transcripts of integrators and key genes believed to be involved in the regulation by light and/or hormones of chlorophyll biosynthesis were analyzed by both the RNA-Seq and RT-PCR (Supplementary Tables S4, S5 and Figure 5).

PHYA and CRY1 sense far-red/red and blue/UV-A light, respectively, and initiate intracellular transduction to alter the expression of nuclear genes (Chaves et al., 2011; Burgie and Vierstra, 2014). Both *PHYA* and *CRY1* increased significantly in the shaded leaves when compared with the non-shaded leaves. Simultaneously, key integrators *EIN3* and *DELLAs* showed significant increases in response to shade treatment. Especially *DELLAs*, including *DELLA1*, *DELLA3*, and *DELLA4* were found to be shade-induced. The E3 ligases EBF1 and EBF2 were found to function with COP1 in EIN3 degradation and ethylene signal regulation of seedling photomorphogenesis (Shi et al., 2016; Wang et al., 2019). Both *EBF1* and *EBF2* showed a response to shade, *EBF1* expression increasing significantly throughout the shade period while *EBF2* expression increased only at 7d. In contrast, as the important integrator of light-hormone signal networks, *HY5* showed a clear decrease in gene expression throughout shade treatment. *GUN4* has been reported to be light-activated and acts as a putative target of HY5 (Lee et al., 2007) to promote the conversion of ALA to the “chlorophyll branch” (Tanaka et al., 2011). No significant shade response was determined in the expression of *GUN4*. Light signal components, including PIFs (PIF1/3), COP1, Far-red elongated hypocotyls (FHYs), and Fluorescent in blue light (FLU) were detected in both the control and shade treatments, but there were no significant changes.

### Correlation Network Between Genes and Metabolites in Response to Shade

**FIGURE 6:**
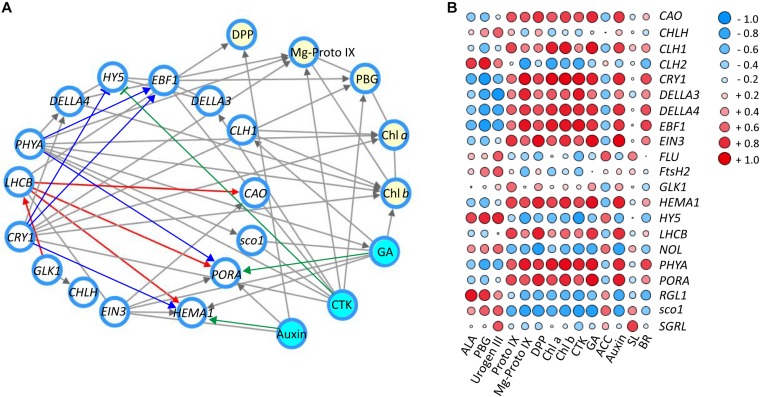
The correlation of genes/TFs, chlorophylls and main precursors and hormones involved in chlorophyll biosynthesis in response to shade treatment. **(A)** Interaction network of main genes and TFs (white nodes), main compounds (yellow nodes) and hormones (blue nodes) involved in chlorophyll biosynthesis (relevant factor ≥ 0.85). **(B)** Correlation heatmap between genes and TFs, main compounds and hormones involved in chlorophyll biosynthesis (-1∼0, expression of genes are negatively correlated; 0, expression of genes are not correlated; 0∼1, expression of genes are positively correlated). Data shown are conducted of three independent biological replicates (*n* = 3), analyzed by the Speaman test in SPSS 13.0 software (IBM SPSS Software, https://www.ibm.com/analytics/datascience/predictive-analytics/spss-statistical-software). The network was conducted by Cytoscape (version 3.7.0; http://www.cytoscape.org/) (Shannon et al., 2003). The heatmap was visualized by the pheatmap package implemented in R (https://cran.r-project.org/web/packages/pheatmap/index.html). For abbreviations, see text.

To further explore the regulatory mechanism by light and hormones on chloroplast development and chlorophyll biosynthesis, the correlation network among expression of candidate genes/TFs, major metabolites in the chlorophyll biosynthesis pathway and hormones were statistically analyzed (Figure 6; Shannon et al., 2003). As shown in Figure 6A, the key genes and TFs involved in the chlorophyll biosynthesis were selected and the interaction network was built between genes and key candidate TFs (relevant factor ≥ 0.80 was selected for the interaction network) (Zhang S. et al., 2018). Significant correlations were detected between blue/red light receptors (*CRY1* and *PHYA*) and the TFs *DELLAs* (*DELLA3* and *DELLA4*), *EFB1* and *sco1*, which are relevant to light signal transductions. Correlations were also found among *CRY1/PHYA* and genes (*PORA* and *HEMA1*) and hormones (CTK and GA) involved in chlorophyll biosynthesis. Strong interaction was detected between the expression of *GLK1* and *LHCB*, suggesting a regulation between these genes in tea leaves. Correlations were also detected among *LHCB* and enzyme encoding genes in the chlorophyll biosynthesis pathway, including *HEMA1*, *PORA*, and *CAO*. Furthermore, HY5 has been known to participate in the light and hormone regulation of chlorophyll biosynthesis (Waters and Langdale, 2009; Xu et al., 2016; Liu X. et al., 2017). In the current study, there was a significant association between the expression of HY5, blue/UV-A light receptor CRY1 and the light signal integrators (*DELLA3/4* and *EBF1*). In addition to the interaction between candidate genes and TFs, the interactions between genes/TFs and relative plant hormones with responses to shade were also conducted. A strong interaction between auxin and HEMA1 expression was detected, consistent with previous findings in *Arabidopsis* (Tanaka et al., 2011; Kobayashi et al., 2012). The hormone GA exerted a regulatory effect on *PORA* expression in tea leaves in response to shade treatment. A strong interaction was also detected between CTK and *HY5*, *PHYA*, *CRY1*, *EBF1*, and *DELLA3*, suggesting some regulation by CTK of chlorophyll biosynthesis in tea leaves. The changes in metabolites involved in chlorophyll biosynthesis (DPP, Mg-Proto IX, PBG, Chl *a*, and Chl *b*) were analyzed, and strong interactions among these metabolites, genes/TFs and hormones were detected (Figure 6A).

To complement the interaction network, a matrix correlation analysis was conducted between genes/TFs and hormones and the major metabolites in the chlorophyll biosynthesis pathway (Figure 6B). Consistent with the information shown in the interaction network, strong positive and negative correlations were detected among integrators of light and hormone signal pathways (*CRY1*, *PHYA*, *HY5*, *DELLAs*, *EBF1*, *EIN3*, *sco1*, and *LHCB*), enzyme encoding genes in chlorophyll biosynthesis (*CAO*, *CHL1/2*, *HEMA1*, *NOL*, and *PORA*), major metabolites of the chlorophyll biosynthesis pathway (PBG, Mg-Proto IX, Chl *a*, and Chl *b*) and plant hormones (auxin, GA and CTK). Furthermore, most of these light- and hormone signal integrators (*CRY1*, *PHYA*, *DELLAs*, *EBF1*, and *EIN3*) showed a positive correlation with the accumulation of chlorophylls and metabolites (Proto IX, Mg-Proto IX, Chl *a*, and Chl *b*). In contrast, *HY5* and *sco1* were found to have a negative correlation with some metabolites (Proto IX, Mg-Proto IX, Chl *a*, and Chl *b*) and hormones (CTK and GA). This may indicate a negative regulation of HY5 and sco1 on these metabolites and hormones in the leaves under shade conditions. *GLK1*, which is reported to play a critical role in light regulation of chloroplast biogenesis in *Arabidopsis* (Waters et al., 2009). *GLK1* showed some positive correlation, at the transcriptional level, with many the metabolites (Urogen III, Proto IX, DPP, and Chl *a*/*b*) and hormones (CTK, GA, and auxin) quantified in this study. For instance, some positive correlation was detected between *GLK1* and Proto IX. Conversely, the changes of ACC, the ethylene precursor was found to be negatively correlated with *GLK1* expression.

## Discussion

Tea is a typical woody crop that prefers environments with a relatively low level of light intensity. Compared to other plants, *Camellia* tea plants can maintain good growth and condition for a long period even in an extremely low light environment. For example, when nearly 98% of the light was excluded by the shading nets, plants still grew well with new bud burst and chlorophylls concentrations increased after a 1 month treatment period (Yang et al., 2012). With such low-light tolerance, shading of plants has been widely used in tea plantations for decades, particularly for green tea with its high chlorophyll content requirement (Yamaguchi and Shibamoto, 1981; Shimoda et al., 1995). However, the molecular mechanism of chloroplast development, photosynthesis and chlorophyll biosynthesis underlying the shade effects remained largely unknown. From the current study, it is proposed that the shade-increased accumulation of chlorophylls in *C. sinensis* cv. Shuchazao leaves is the result of at least two factors: (a) positive changes in chloroplast development and (b) a light regulation of genes and potential TFs involved in the chlorophyll biosynthesis pathway. The changes of hormones, as a part of shade responses, presumably synergize with light to regulate chlorophyll biosynthesis in the tea crop.

### Shade Expanded the Photosynthetic Apparatus

The biogenesis of a mature chloroplast is a complex and sophisticated process. Numerous genes and signal integrators participate in this process and together with light play crucial roles in the transition from a proplastid to a mature chloroplast (Waters and Langdale, 2009; Liu X. et al., 2017). Compared with findings in the model plant *Arabidopsis* (Lichtenthaler et al., 1981), similar responses to shade were observed in the leaves of Shuchazao. In the current study, the reduction of light stimulated the chloroplast development quantitatively (Figure 1). These changes included the abundance of chloroplasts, ultrastructural changes in the thylakoid membrane system and the formation of grana stacks. These were consistent with responses to shade reported in previous studies (Wu et al., 2016; Liu G. F. et al., 2017), with similar alterations in chloroplast ultrastructure observed in both the albino tea cultivars and yellow phenotype.

**FIGURE 7:**
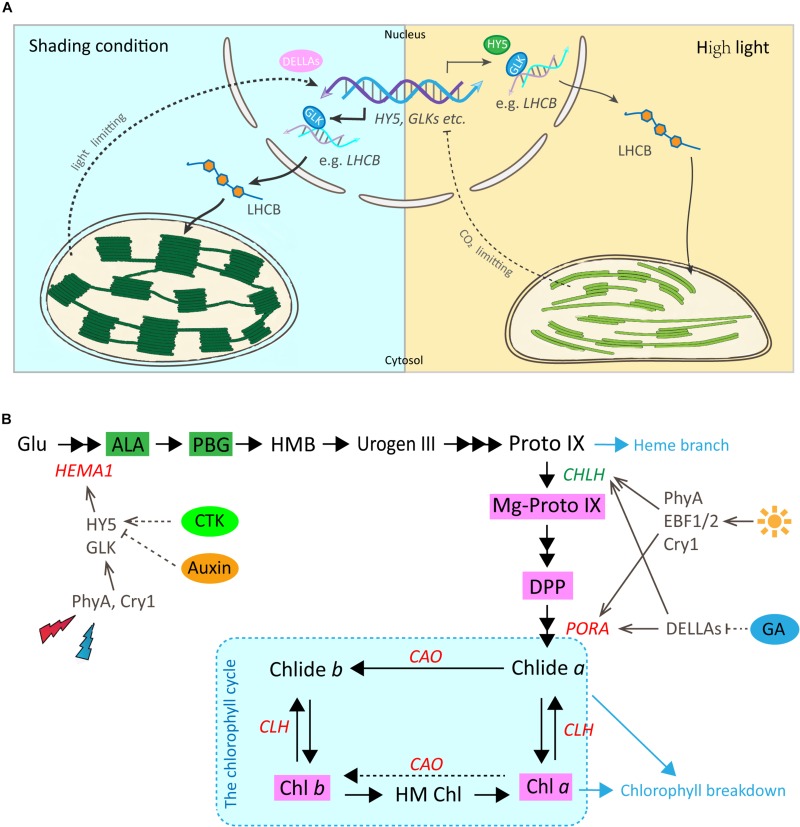
Working models for the regulatory roles of light and hormones in chloroplast development and chlorophyll biosynthesis in Shuchazao leaves**. (A)** A predicted model for the regulatory role of light on chloroplast development by the GLK and other potential TFs in tea leaves (reproduced, in part, with permission from Waters and Langdale, 2009). Under the shade environment, the photosynthetic electron transfer chain is in an oxidized state and cannot provide sufficient ATP. Therefore, a light-limitation signal from the chloroplast is sent to the nucleus and GLK genes are up-regulated. Transcript levels of these GLK target genes increase leading to higher levels of proteins that function in light-harvesting (such as LHCB, etc.), and, therefore, assembly leads to higher specific chlorophyll levels, a lower Chl *a*/*b* ratio and more abundant grana. In contrast, when light is plentiful and the rate of CO_2_ fixation is insufficient, this prompts a chloroplast-derived CO_2_ fixation limiting signal to the nucleus. The transcriptions of GLK genes are repressed and the accompanying decrease in LHCB and chlorophyll-related gene transcripts, eventually results in a reduction in the thylakoid membrane and lower chlorophyll content. **(B)** A simplified regulatory network of chlorophyll biosynthesis by light and plant hormones in tea leaves (Red, increase in response to shade; Green, decrease in response to shade). The dashed portion indicates that further verifications are needed. For abbreviations, see text.

In *Arabidopsis*, a model for light regulation on chloroplast development has been summarized according to previous studies (Figure 7A, this model was modified from Waters and Langdale, 2009). Nuclear TF GLKs are required for transcription of genes encoding chloroplast proteins and provide the anterograde signal in response to light regulation (Oh and Montgomery, 2014). Simultaneously, LHCB acts as a bridge passing this anterograde signal from the nucleus to chloroplast to regulate the formation of the thylakoid membrane and grana development (Waters and Langdale, 2009). The data from the current study suggest that the regulation on chloroplast development by light in Shuchazao leaves is comparable to that of *Arabidopsis*. Compared with non-shaded leaves, an increase in the expression of *GLK1* was detected immediately in the shaded leaves. *LHCB*, the central signal bridge between the nucleus and chloroplast, showed a significant increase in the shaded leaves throughout the whole treatment period (Figure 3). Also, strong correlations were detected between the expression pattern of *LHCB* and the shade responses of downstream genes, *PORA*, *HEMA1*, and *CAO* (Figure 6). These findings are consistent with the model that *GLK* genes are up-regulated once the nucleus receives a light-limiting signal derived from the chloroplast, and, therefore, leading to higher expression of its target gene *LHCB* (Waters and Langdale, 2009; Oh and Montgomery, 2014). In addition, DELLAs stimulates photosynthesis and chlorophyll biosynthesis through regulating *LHCB*, *CHLH*, and *PORA*/*B*/*C* (Cheminant et al., 2011). Our data showed the expression of DELLAs (*DELLA3* and *DELLA4*) became activated in the shaded leaves (Figure 5). HY5 is supposed to be involved in the anterograde signal perception. Previous studies suggested that HY5 acts as a signal promoter in the light and hormone signaling pathways in regulating nuclear gene transcription and promoting seedling photomorphogenesis (Chen et al., 2004; Casal et al., 2014; Xu et al., 2016). Several nuclear encoding photosynthetic and chlorophyll biosynthesis genes, such as *CHLH*, *GUN4*, *PORC*, *CAO*, and *CHL27*, are the putative targets of HY5 (Lee et al., 2007). Recent findings suggest HY5 mediates the process of chlorophyll biosynthesis in plant roots and functions downstream of PHYB to modulate *PORA* expression through a Myb-like transcription factor REVEILLE1 (RVE1) (Jiang et al., 2016). Herein we found that the transcriptional level of *HY5* decreased significantly in leaves under the shade conditions (Figure 5). This decrease was probably due to the heavy limitation of light exposure (red, blue, and UV-B signals) under shading conditions, which overlays the intrinsic needs of chloroplast development in the opposite way.

### Shade Stimulates the Gene Activities Involved in Chlorophyll Biosynthesis

In addition to the physical enhancement of the photosynthetic apparatus (thylakoid membrane systems) as shown in the ultrastructure alterations, metabolites and gene activities in the chlorophyll biosynthesis pathway showed a clear light regulatory role (Figure 7B). Significant increases were detected in the level of major metabolites, including My-Proto IX, DPP, Chl *a*, and Chl *b* (Figure 2). The accumulation of My-Proto IX increased immediately at 1d after commencing shade treatment, while the DPP was seen to increase at the later stages of shade treatment. The accumulation of Chl *a* and Chl *b* presented significant increases (around 2 x) in shaded leaves (Figure 2C), with a decrease in the ratio of Chl *a*/Chl *b* (Figure 2B).

These can be explained by the changes in the activities of genes and TFs in chlorophyll biosynthesis (Figure 4). *PORA*, the well known light-regulated gene (Reinbothe et al., 1996), showed a clear up-regulation throughout the shade period. The transcript level of *CAO* increased significantly in the shaded leaves, especially at the later stage of the shade from 3d to 14d. A similar increase was detected in the expression of *CLH1*, which increased to nearly 2x at 14d of shade treatment. In addition, light receptors and transcription factors that are believed to function in the chlorophyll biosynthesis pathway were also investigated. Previous studies reported HY5 plays a vital role in the convergence of blue, red and far-red light-signal pathways for regulating the transcription level of *HEMA1* (McCormac and Terry, 2002). In the present study, we found a significant reduction of *HY5* expression as discussed earlier, but a significant increase in the expression of *HEMA1*. Also, both the red/far-red light sensor *PHYA* and blue light receptor *CRY1* showed clear increases. These findings were consistent with the previous studies, implying a regulation of red/far-red and blue light signals on *HEMA1* expression through *HY5* (McCormac and Terry, 2002). Furthermore, light signal components, EBFs and DELLAs were detected to be light-regulated, suggesting a role of light regulation in tea leaves consistent with previous studies (Cheminant et al., 2011; Shi et al., 2016). Interestingly, *GLK1*, which was expected to have a strong correlation with the chlorophyll biosynthesis pathway, presented no significant connection with the metabolites measured in this study. It appears that *GLK1* functioned in the light regulation of chloroplast development rather than chlorophyll biosynthesis in tea leaves. Further analysis needs to be carried out at the protein level to investigate the role of *GLK1* in the tea leaves. In total, all of these data show the chlorophyll biosynthesis pathway, in particular, the “chlorophyll cycle” became much more active under the shade conditions to meet the requirements of photosynthetic pigments and energy needs.

### The Involvement of Hormones in the Regulation of Chlorophyll Biosynthesis

Multiple hormone pathways may participate in mediating the shading responses in tea plants. HY5 is a key light signal component that integrates the light- and hormone signal pathways, including auxin, CTK, abscisic acid and GA (Lau and Deng, 2010). Previous studies have shown that auxin and CTK regulate *HY5*, at the protein level, to promote seedling photomorphogenesis (Lau and Deng, 2010). Conversely, HY5, together with its close homolog HY5 Homolog (HYH), repress auxin signaling by direct activation of its negative regulators Indole Acetic Acids 7 and 14 (IAA7 and IAA14) (Cluis et al., 2004; Sibout et al., 2006). In the present study, the transcription level of *HY5* was decreased, simultaneously significant increases were detected in the level of CTK, GA and auxin under shade conditions (Figure 5 and Table 1). Also, strong negative correlations were detected between HY5 expressions and the level of hormones, CTK (relevant factor -0.86) and auxin (relevant factor -0.65) (Figure 6). Therefore, our data support the theory that HY5 negatively modulates auxin signaling to mediate responses to shade in tea plants. *HEMA1* is one of the target genes of HY5 (McCormac and Terry, 2002) and the expression of *HEMA1* is potentially regulated by both auxin and CTK signals in tea leaves as parts of the shade response (relevant factor between *HEMA1* and auxin is 0.7; relevant factor between *HEMA1* and CTK is 0.85). In addition DELLAs, a subfamily of the GRAS transcriptional regulators (Jiang and Fu, 2007), have been suggested as key nuclear-enriched repressors in the GA signaling pathway, which represses GA-regulated gene expression and seedling growth (Jiang and Fu, 2007; Cheminant et al., 2011). Also, DELLAs positively regulate the expression of genes involved in chlorophyll biosynthesis (*CHLH* and *PORC*) in a PIF dependent manner (Cheminant et al., 2011), and independently up-regulate photosynthesis (*LHCB*) and *PORA/B* gene expression. However, in our data, both the GA and *DELLA3* showed significant increases after shade treatment. Further studies can focus on the roles of CTK and GA signaling in mediating responses to shade in *Camellia sinensis*. Overall, based on the data generated in the current study, it is presumed that hormone changes, as a part of the shade response, may play some role in modulating chlorophyll biosynthesis in the tea leaves.

## Conclusion

This study aimed to investigate the molecular mechanism underlying the shade-enhanced accumulation of chlorophylls in an important tea cultivar, *C. sinensis* cv. Shuchazao. Based on the data from the transcriptional expression of genes/TFs, alterations of metabolites and hormones, we proposed that the shade-enhanced accumulation of chlorophylls in Shuchazao leaves is modulated by a light-hormone network, functioning from ultrastructural construction of chloroplasts to gene activities of the chlorophyll biosynthesis pathway. Our findings showed the light-hormone mediated regulatory network on chlorophyll biosynthesis in tea leaves, which improves our understanding of the regulatory mechanism on chlorophyll biosynthesis and could provide new opportunities for the molecular breeding of tea cultivars with specific phenotypes.

## Data Availability Statement

The datasets generated for this study can be found in the NCBI accession PRJNA576575.

## Author Contributions

LL conducted this research, analyzed the data, and prepared the draft manuscript with assistance from NL, XL, SY, and WW for samplings and data analysis. XW provided supervision for the research. All of the authors read and approved the final manuscript.

## Conflict of Interest

The authors declare that the research was conducted in the absence of any commercial or financial relationships that could be construed as a potential conflict of interest.

## Footnotes

1 http://tpia.teaplant.org

2 https://www.ncbi.nlm.nih.gov/tools/primer-blast/

## Abbreviations

ACC: 1-aminocyclopropane carboxylic acid
AHK: Arabidopsis Histidine Kinase
ALA: 5-aminolevulinic acid
ALAD: ALA dehydratase/PBG synthase
ANOVA: one-way analysis of variance
ARCs: accumulation and replication of chloroplasts
ARR: the type B Arabidopsis response regulators
BR: brassinosteroid
BRM: BRAHMA
CAO: chlide a oxygenase
CBR: Chl *b* reductase
Chl *a/b*: chlorophyll *a/b*
CHL: magnesium chelatase encoding gene
CHLG: Chl synthase
CHLH: magnesium chelatase
CLH: chlorophyllase
ClpP6: Clp protease proteolytic subunit 6
COP1: Constitutively Photomorphogenic 1
Cpn60: heat shock protein family D (Hsp60) member 1
CPO: coprogen III oxidase
CRYs: cryptochromes
CTK: cytokinin
DELLAs: gibberellic acid-regulated growth-repressing DELLA proteins
DET1: de-etiolated 1
DPP: divinyl protochlorophyllide
DVR, 3: 8-divinyl pchlide a 8-vinyl reductase
EBF1/2: EIN3-binding F-box 1/2
EIN3/EIL1: Ethylene Insensitive 3/EIN3-LIKE 1
ELISA: enzyme-linked immunosorbent assay
ETH: ethylene
EX1/2: EXecuter1/2
FAR: FAR-red-impaired response
FeCh: ferrochelatase
FHY: FAR-RED Elongated HYpocotyl
FLU: FLUorescent in blue light
FPKM: fragment per kilobase of exon model per million mapped reads
FtsH2: filamentation temperature-sensitive H2
FtsZ1/2: tubulin-like protein FtsZ1/2
GA: gibberellins
GC1: giant chloroplast 1
GLK: golden2-like protein
GluRS: Glu-tRNA synthetase
GluTR: Glu-tRNA reductase
GSA-AT: GSA aminotransferase
GUN4: GENOMES UNCOUPLED 4
HCAR: 7-hydroxymethyl Chl *a* reductase
HDA15: histone deacetylase 15
HEMA: Glu-tRNA reductase
HO: heme oxygenase
HSP: heat shock proteins
HY2, phytochromobilin: ferredoxin oxidoreductase
HY5: Elongated HYpocotyl 5
HYH: HY5 Homolog
IAA: Indole-3-Acetic Acid
LHCB: light-harvesting complex II chlorophyll binding protein
LPA2: Low PsII Accumulation 2
LSD: least significant difference
MgCh: magnesium chelatase
MgCY: Mg-Proto IX monomethyl ester cyclase
MgD: Mg-dechelatase
MgMT, S-adenosyl -L-methionine: Mg-Proto IX methyltransferase
Mg-Proto IX: Mg-Protoporphyrin IX
MinD: chloroplast division factor MinD
MSL2: MscS-like/mechanosensitive ion channel protein 2
NOL: Chl *b* reductase encoding gene NOL
PAO: pheophorbide a oxygenase
PAT1: scarecrow-like transcription factor PAT1
PBG: porphobilinogen
PBGD: PBG deaminase/HMB synthase
PDV1/2: ARC5 recruitment factor PDV1/2
PET: photosynthetic electron transport
PHYs: phytochromes
PIF: Phytochrome Interacting Factor
POR: pchlide oxidoreductase
PPH: pheophytinase
PPO: protoporphyrinogen IX oxidase
Proto IX: protoporphyrin IX
PTM: PHD type transcription factor with transmembrane domains
RCCR: red Chl catabolite reductase
RGL1: DELLA protein RGL1
rpoT: NEP protein encoding gene
RT-PCR: quantitative real-time PCR
RVE1: REVEILLE 1
SAL1: chloroplast retrograde signal gene SAL1
sco1: snowy cotyledon 1
SL: strigolactones
STY, serine: threonine and tyrosine
TEM: transmission electron microscope
TFs: transcription factors
THF1: thylakoid formation 1
TOC: translocase of chloroplast
TRX: thioredoxin
UROD: urogen III decarboxylase
Urogen III: uroporphyrinogen III
UROS: urogen III synthase
VIPP1: vesicle inducing plastid protein.
